# Assessment of the Prevalence of Infections in Pediatric Patients With Acute Lymphoblastic Leukemia

**DOI:** 10.7759/cureus.46837

**Published:** 2023-10-11

**Authors:** Arun Nair, Ruaa Elballushi, Riecha Joshi, Sanvithi Anjanappa, Maksuda Akter, Sehrish Arif, Sana Rehman

**Affiliations:** 1 Pediatrics, Saint Peter’s University Hospital, New Brunswick, USA; 2 School of Medicine, Royal College of Surgeons in Ireland - Medical University of Bahrain, Busaiteen, BHR; 3 Pediatrics, Government Medical College, Kota, IND; 4 School of Medicine, Kempegowda Institute of Medical Sciences, Bangalore, IND; 5 School of Medicine, American International Medical University, Gros Islet, LCA; 6 Medicine, Fatima Memorial Hospital College of Medicine and Dentistry, Lahore, PAK

**Keywords:** antibiotic stewardship, fungal infections, viral infections, bacterial infections, pediatrics, acute lymphoblastic leukemia

## Abstract

Infections cause notable treatment-related morbidity during pediatric acute lymphoblastic leukemia/lymphoma (ALL/LLy) therapy. Infections are the most critical cause of morbidity and mortality in children undergoing treatment for acute lymphoblastic leukemia (ALL). Children with ALL, who are frequently underweight, are at increased risk of community-acquired pathogens, nosocomial multidrug-resistant pathogens, and opportunistic microorganisms. A weakened immune system from ALL itself and chemotherapy's side effects further worsen the prognosis. PubMed and Google Scholar articles were curated in a Google document with shared access. Discussion and development of the paper were achieved over Zoom meetings. This narrative review aims to analyze and summarize various pathogens responsible for infections in children receiving treatment for ALL and their treatment regimen and prophylaxis. The incidence of viral infection is higher in ALL patients, followed by bacterial and fungal infections. Prevention via prophylaxis and timely initiation of treatment is essential for positive outcomes.

## Introduction and background

Acute lymphoblastic leukemia (ALL) refers to the malignancy of lymphocytes (B or T lymphoblasts) because of the uncontrolled monoclonal proliferation of lymphoid cells. The abnormal rapid growth of immature lymphocytes results in the expanded invasion of the bone marrow, blood, and other extramedullary organs. According to the World Health Organization, ALL can be categorized into two main subtypes based on lymphoid neoplasms: B cell acute lymphoblastic leukemia and lymphoblastic lymphoma (B-ALL/LBL) and T cell acute lymphoblastic leukemia and lymphoblastic lymphoma (T-ALL/LBL). Once the precursor cells expand to the blood and bone marrow, the manifestation can be classified as leukemia. Whereas if the expansion reaches extramedullary organs and tissue, the manifestation is classified as lymphoma. ALL is the most common malignancy among children, accounting for 25% of all pediatric cancers [1]. ALL is often diagnosed between the ages of two and ten, with the first peak between the ages of two to five years. It is more common in children with genetic disorders such as Bloom syndrome and Down syndrome. Pediatric ALL's outcome has significantly improved, with 90% of children witnessing a five-year survival rate improvement in 2009 compared to a 20% lower survival rate in 1974 [2]. Although the exact etiology of ALL remains unknown, several risk factors have been reported to be strong predictors of ALL in young children. Risk factors can be conservatively divided into three broad categories. Individuals with specific congenital syndromes are at an increased risk of developing ALL. Down syndrome, Ataxia telangiectasia, bloom syndrome, Fanconi anemia, and Nijmegen breakage syndrome are associated with increased ALL cases in children. Klinefelter, Neurofibromatosis, and Li-Fraumeni syndromes have also been associated with ALL in young children [3]. Congenital disorders are associated with poor outcomes and higher relapse rates after treatment. Environmental toxins have been determined to be a strong predisposing factor to most pediatric cancers. This applies to the exposure of the mother during pregnancy or directly to the child during infancy. Such toxins include hydrocarbon (found in gasoline and paint thinners) exposure, pesticide exposure, ionizing radiation, benzene, and nicotine inhalation [4]. Childhood infections and other predictive factors such as viral infections can cause permanent changes to the child's DNA. DNA methylation results in abnormal gene expression that eventually progresses to the development of ALL [5]. Although infections generally have not shown a correlation with the development of ALL within the first year of life, infections acquired afterward have shown a strong correlation. Parvovirus B19 is particularly linked to ALL development, as children infected with this virus seem to be at an increased risk of developing ALL shortly after [6]. Furthermore, human T-cell lymphoma/leukemia virus-1 (HTLV-1) and Epstein-Barr virus (EBV) can also cause a rare form of ALL. HSV, CMV, and Influenza have also been seen to be associated with ALL. High birth weight can coincide with abnormal gene expression of insulin growth factor (IGF), which can be a predictive factor of ALL [7]. 

Signs and symptoms of ALL are those associated with changes in hematopoiesis and can be grouped into anemia and its associated symptoms, thrombocytopenia, bleeding-associated symptoms, and finally, granulocytopenia. Granulocytopenia makes patients more susceptible to infections, especially bacterial infections resulting in extreme pyrexia. Young children might initially present with flu-like symptoms from infection; however, blood tests would reflect abnormally alarming numbers that would suggest an underlying cause. Diagnosis is frequently made on an average of two weeks from initial symptom presentation through complete blood count, bone marrow examination, blood smear, and histochemical testing. Immunophenotyping and cytogenetics can also be used to determine ALL types. All are traditionally treated through chemotherapy and managed through supportive care. Children with ALL are often started with induction therapy right away which involves a combination of treatment with corticosteroids, vincristine, 1-asparaginase, and anthracycline [8]. After initial treatment, maintenance therapy has been recently adopted to maintain remission in young children by prescribing 6-mercaptopurine (or methotrexate) orally once a month. ALL treatment averages between two to three years. Depending on the type of ALL, different staging, and response to treatment, treatment protocols are often modified in style, length, and combination. For instance, in Ph+ ALL (Philadelphia positive Acute Lymphoblastic Leukemia), the tyrosine kinase inhibitor is combined with systemic chemotherapy to achieve remission. Although treatment for pediatric ALL is considered to have a high survival rate for children, infections while on chemotherapy are, unfortunately, the leading cause of morbidity and mortality in children with ALL. For instance, hospital-acquired pneumonia is fatal to children with ALL if intervention is not fast and effective. Therefore, early intervention is of absolute importance to prevent complications and ensure the safety and well-being of the child while undergoing chemotherapy. In this study, we explore the different types of infections that a patient with ALL is prone to get and discuss a few factors to help with early intervention. 

## Review

Methods

A literature review format was used to assess pre-existing research articles on ALL to understand the prevalence of infections in ALL patients, the risk factors, the rate of infections, the chemotherapy associated with it, and the suggestions to prevent complications. Only the articles published after 2014 were considered in the study. Extensive searches and analyses were done from multiple PubMed and Google Scholar databases. Keywords used in identifying relevant articles include "pediatric acute lymphoblastic leukemia", "fungal infections", "bacterial infections", "viral infections", "antimicrobial resistance", and "clinical outcomes in pediatric ALL". The data were confirmed by the multiple investigators involved in the review.

Infections in ALL

Bacterial Infections

Gram-positive (62%) bacterial infection was more prevalent in patients undergoing HSCT, and coagulase‐negative staphylococci were most commonly encountered. Fluoroquinolones, a common prophylactic antibiotic, were not used to lower the burden of Gram-Negative strains, given the resistance to it being frequent [10].

It has been observed that in patients undergoing chemotherapy, levofloxacin (fluoroquinolone) was responsible for reducing bacterial infection (15.9%) as compared to no prophylaxis (37%), including Clostridium difficile (0%) as compared to no prophylaxis (9.8%) [10] (Table 1). In another study, children receiving chemotherapy significantly reduced bacteremia with levofloxacin prophylaxis. However, no significant reduction was seen in patients undergoing HSCT for the same [11]. In China, fluoroquinolones are not allowed in children; thus, beta-lactamase antibiotics and G-CSF after achieving remission and intravenous immunoglobulin are used as prophylaxis, which showed a reduction in septicemia [12].

**Table 1:** Outline of implicated bacterial infections identified in pediatric ALL patients ALL: Acute lymphoblastic leukemia

Bacteria	Rate of Infection	Chemo	Antimicrobial prophylaxis	Additional comments	Reference
Staphylococcus epidermidis	22%	Allogeneic hematopoietic stem cell transplantation	Rooms with reverse isolation and high‐efficiency particulate air filters during the neutropenic period and received a low bacterial diet. amphotericin B and azoles	Transplant from a matched unrelated donor was found to be associated with increased risk of severe infections given the use of intensified Graft versus host disease prophylaxis that consisted of Anti-thymocyte globulin (ATG) and methotrexate	[9]
Staphylococcus haemolyticus	11%				
Other coagulase‐negative staphylococci	9%				
Enterococcus spp.	16%				
Streptococcus viridans group	7%				
Escherichia spp.	25%				
Pseudomonas spp	19%				
Enterobacter spp.	9%				
Klebsiella spp	6%				
Clostridium difficile	0	Induction therapy for ALL	Levofloxacin	Prophylaxis prevented febrile neutropenia and systemic infection Levofloxacin prophylaxis also minimized the use of treatment antibiotics and drastically reduced C. difficile infection.	[10]
	9.8%		No prophylaxis		
Bacterial infection	37		No prophylaxis		[10]
	15.9		Levofloxacin		
			Intravenous cefepime alone or vancomycin plus oral cephalosporin, oral ciprofloxacin, or intravenous cefepime		
Bacteremia		Stem cell transplant	Levofloxacin	Levofloxacin prophylaxis did not reduce the risk of bacteremia.	[11]
Septicemia	12.9%	Multi‐agent chemotherapy		For treatment, 2010 IDSA guidelines recommend monotherapy with betalactams, piperacillin, and carbapenem. In case of a resistant strain, addition of vancomycin is advised.	[12]
Coagulase-negative staphylococci	20.1%				
Staphylococcus epidermidis	14.6%				
E. coli	11.5%				
Klebsiella pneumoniae	8%				
Pseudomonas aeruginosa	7%				
Staphylococcus aureus	5.6%				
Streptococcus mitis	3.2%				
Streptococcus pneumoniae	3%				
salmonella	2%				
Febrile neutropenia (causing increased risk of infections, especially bacterial)		Vincristine and dexamethasone (as a replacement for prednisone) pulses in continuation phase of chemotherapy	Routine antifungals, antibacterials, and colony stimulating factors were NOT administered. Patients were advised to wear a particulate filtration mask during induction and re-induction phases	Dexamethasone decreases ANC and increases risk of infections.	[13]
Sepsis		Dexamethasone in induction therapy	N/A	Substitute dexamethasone with prednisone to reduce the incidence of sepsis.	[13]
Any bacterial, viral, or fungal infections		CD19-targeted chimeric antigen receptor–modified T (CAR-T) cells administered after lymphodepletion chemotherapy	Acyclovir 800 mg or valacyclovir 500 mg twice a day for herpes simplex or varicella zoster virus seropositive individuals starting on the day of lymphodepletion until ≥3 months after CAR–T-cell infusion, levofloxacin 750 mg daily and fluconazole 400 mg daily while the ANC was <500 cells per mm3, and trimethoprim 160 mg/sulfamethoxazole 800 mg	Incidence of infection was higher in patients who received a higher CAR–T-cell dose, and high CAR–T-cell doses are associated with an increased risk of CRS and neurotoxicity Infections in adult patients with relapsed B-ALL are common after CD19 CAR T-cell therapy. Understanding the infectious complications that are temporally coincident with CD19 CAR T-cell therapy is critical for developing effective prophylactic and other supportive care measures to improve clinical outcomes.	[14,15]

Replacement of dexamethasone with prednisone in the induction and continuation phase of chemotherapy has been found to reduce rates of febrile neutropenia and reduce the incidence of septicemia [13]. Close monitoring and some modification in chemotherapy can be explored as an addition to prophylactic antibiotics to prevent bacterial infections.

Immunotherapy with CD19-targeted chimeric antigen receptor-modified T (CAR-T) cells is a novel treatment for patients with relapsed or refractory B-cell malignancies, including ALL. Because patients are exposed to prior chemotherapies, their immunity is poor. Thus, they are more prone to infections. Antibiotic prophylaxis and IV immunoglobulin repletion have effectively prevented and treated infection [14].

Viral Infections

The cumulative incidence of infections was the highest for viruses compared to bacterial and fungal infections. The most critical viruses responsible for infections in patients after HSCT are EBV, cytomegalovirus (CMV), and adenovirus (AdV). In a study conducted in 2017, screening was done for these three viruses every week starting the day after the transplant. If the screening was positive, CMV was treated with ganciclovir, EBV with rituximab, and AdV with cidofovir. CMV prophylaxis is especially required for transplant recipients with a seropositive CMV IgG. Ganciclovir has been found effective as a prophylactic agent for CMV reactivation [9].

Although non-fatal in the average population, influenza A and B viruses can be lethal in ALL chemotherapy patients (Table 2) [16]. Neuraminidase inhibitors such as oseltamivir and zanamivir have effectively treated the same. Although preventive vaccination is considered to be the best way to prevent Influenza-associated illness, it was found in a study that the use of trivalent inactivated influenza vaccine (TIV) didn’t protect against Influenza as compared to unvaccinated individuals, as 71% of the patients receiving the TIV still were reported with Influenza-related illness [17]. Respiratory syncytial virus was adequately treated with palivizumab [16].

**Table 2:** Outline of implicated viral infections identified in pediatric ALL patients HBV: Hepatitis B virus; HCV: hepatitis C virus; RSV: respiratory syncytial virus; EBV: Epstein‐Barr virus; CMV: cytomegalovirus; ALL: acute lymphoblastic leukemia

Virus	Rate of Infection	Chemo	Antimicrobial prophylaxis	Additional comments	Reference
HBV		Anti-CD20/or CD52 therapy	Entecavir, tenofovir, lamivudine, adefovir, telbivudine	Advised to use in combination with other drugs such as adefovir.	[16]
HCV		Transplantation, anti-CD20 therapy	Ledipasvir/simeprevir and sofosbuvir, paritaprevir and ritonavir, ombitasvir and dasabuvir		[16]
HIV		Chemotherapy	Integrase inhibitors, non-nucleoside reverse transcriptase inhibitors	Successfully treated with antiretroviral agents	[16]
Influenza A/B		Chemotherapy	Oseltamivir, zanamivir	Can reduce the duration of uncomplicated influenza A and B illness by approximately 1 day when administered within 48 hours of illness onset compared with placebo	[16]
	73%	TOTALXVI protocol	Trivalent Inactivated Influenza Vaccine	Trivalent inactivated influenza vaccine had inadequate protection compared to unvaccinated individuals	[17]
RSV		Chemotherapy	Palivizumab,	Resistance to palivizumab rare but possible	[16]
Norovirus		HSCT	Ribavirin, Interferons, Immunoglobulins	A need for a vaccine has been described in the literature for this virus	[16]
		HSCT	Nitazoxanide		[18]
Adenovirus		Chemotherapy	Cidofovir	No FDA-approved treatments for adenovirus have been described but there are reports of Cidofovir being used in immunocompromised patients	[16]
EBV	24%	Allogeneic hematopoietic stem cell transplantation	Rooms with reverse isolation and high particulate matter air filters required during periods of neutropenia. If screening tests for these viruses were positive, patients received ganciclovir, cidofovir or rituximab, respectively as prophylaxis.	Transplantation associated with increased risk for any time of infections in the pre-engraftment period and for viruses, typically between day 30 and day 100 after transplantation which may be explained by intensified graft versus host disease prophylaxis and subsequent administration of methotrexate	[9]
CMV	17%				
BKV	20%				
Adenovirus	14%				
Others such as Varicella and various Herpes	28%				
Modest risk: CMV, PJP, HBV reactivation		BCR-ABL TK inhibitors (imatinib, dasatinib, nilotinib, bosutinib, ponatinib)	No clear benefit from routine prophylaxis Screen for HBV infection	Avoid with azole Monitor QTc	[19]
HSV, VZV, CMV, PJP, PML, fungal per NCCN		Anti-CD19 bispecific T-cell engager (blinatumomab)	ACV and PJP prophylaxis		[19]
COVID 19	CAR-T cell therapy	CAR-T cell therapy	NA		[20]
RVI	HSCT	HSCT	NA		[21]
H1N1		chemotherapy	1: Broad-spectrum antibiotics (meropenem and vancomycin) and antifungal drug (liposomal amphotericin 2: Broad-spectrum antibiotics (cilastatin, imipenem, and vancomycin), an antifungal drug (liposomal amphotericin B) and granulocyte colony growth factor were administered. Additionally, intravenous immunoglobulin was also administered		[22]

Norovirus is an important non-bacterial cause of gastroenteritis in immunocompromised patients due to its ability to break down mucosal barriers and cause sepsis. It is uncertain how beneficial ribavirin, immunoglobulins, and interferon have been in preventing infection. Thus, further research is required to find an effective vaccine for the same. Nitazoxanide is found to be effective in the treatment of Norovirus in patients with HSCT [18].

Immunosuppression in ALL patients undergoing chemotherapy can give rise to the reactivation of various infections, including Hepatitis B virus. Lamivudine is considered effective as a prophylactic to prevent reactivation of HBV. However, prolonged use can lead to resistance; thus, a combination with adefovir has been recommended [16]. It was observed that entecavir and tenofovir are less likely to develop resistance, so they can be used as monotherapy; however, the use of both is not indicated unless the viral load is exceptionally high. Cidofovir, a drug with significant toxicity, is still considered important for treatment because of its potent antiviral action. Brincidofovir, which is a lipid conjugate of cidofovir, is found to be less nephrotoxic as compared to the latter, given the fact that it doesn’t get concentrated in the proximal convoluted tubule like cidofovir [18]. Thus, further research is required to see the effects of brincidofovir as prophylaxis. The reactivation of hepatitis C is prevented by prophylaxis using ledipasvir/simeprevir and sofosbuvir, paritaprevir and ritonavir, ombitasvir, and dasabuvir.

In patients undergoing anti-CD19 bispecific T-cell engager (blinatumomab) chemotherapy, Acyclovir has been recommended to prevent Herpes Simplex Virus (HSV) and Varicella Zoster Virus (VZV) infections [19]. AH1N1 infection was found to be effectively treated by oseltamivir, and the study highlighted the importance of early initiation of antiviral therapy and empirical treatment, given the ineffectiveness of seasonal influenza vaccine in immunocompromised patients of ALL [22]. During the COVID-19 pandemic, immunosuppression in ALL patients contributed to increased viremia [20]. It was also seen that the patients who developed COVID during chemotherapy had more significant morbidity due to acute respiratory distress syndrome requiring oxygen therapy and greater mortality (case fatality rate=0.418) [23].

Fungal Infections

The most common fungal infections were Aspergillus spp. (48%) and Candida spp. (38%). Invasive fungal infections (IFIs) are a significant cause of concern in the case of immunocompromised patients undergoing chemotherapy for ALL. It is advised to start prophylaxis when the absolute neutrophil count (ANC) is ≤ 500/ul. It has been observed that posaconazole (18%) reduced infections compared to no prophylaxis (45%) (Table 3). Fluconazole also had an increased infection rate (72%), more than in cases where no prophylaxis was given [24].

**Table 3:** Fungal infections in acute lymphoblastic leukemia

Fungi	Rate of Infection	Chemo	Antimicrobial prophylaxis	Additional comments	Reference
IFA (invasive fungal infection): Candida and Aspergillus	45%		No prophylaxis	Considering that posaconazole suspension formulation is highly variable, all patients took this agent with food to ascertain that patients had adequate exposure. Patients in either group were permitted to receive amphotericin B or other systemic agents as empirical anti-fungal therapy for a suspected invasive fungal infection.	[24]
	18%		Posaconazole		
	72%		Fluconazole		
Pneumocystis pneumonia	5% to 16%	Allogeneic bone marrow transplantation	No prophylaxis		[25,26]
	0.08%		TMP/SMX		
	0% to 1.3%		Pentamidine		[27]
		Chemotherapy	trimethoprim/sulfamethoxazole,dapsone, pentamidine, atovaquone, pyrimethamine, sulfadoxine, and clindamycin and primaquine in combination	1) Consider administering systemic antifungal prophylaxis to children and adolescents with newly diagnosed and relapsed acute lymphoblastic leukemia at high risk for IFD. 2) Do not routinely administer systemic antifungal prophylaxis to children and adolescents with acute lymphoblastic leukemia at low risk for IFD. 3) Do not routinely administer systemic antifungal prophylaxis to children and adolescents with cancer at low risk for IFD, such as most pediatric patients with lymphomas and solid tumors. 4) Administer systemic antifungal prophylaxis to children and adolescents undergoing allogeneic HSCT pre-engraftment and to those receiving systemic immunosuppression for the treatment of graft-versus-host disease (GVHD). 5) We suggest that systemic antifungal prophylaxis not be used routinely in children and adolescents undergoing autologous HSCT. 6) If systemic antifungal prophylaxis is warranted, administer a mold-active agent. 7) In choosing a mold-active agent, administer an echinocandin or a mold-active azole. 8) Do not use amphotericin routinely as systemic antifungal prophylaxis. 9) If systemic antifungal prophylaxis is warranted, consider administration during periods of observed or expected severe neutropenia [17].	[25]
						Aspergillus spp.	48%	Allogeneic hematopoietic stem cell transplantation	rooms with reverse isolation and high‐efficiency particulate air filters during the neutropenic period.	Transplantation from MUD was associated with an increased risk for any type of SI in the pre‐engraftment period, and for viruses between day +30 and + 100 after HSCT. This could be explained by an intensified GvHD prophylaxis consisting of serotherapy with ATG and administration of methotrexate for recipients of MUD transplants	[9]
Candida spp.	38%	Amphotericin B and acyclovir									
Other fungi including mucormycetes	14%										
Aspergillus and Candida		Chemotherapy	Posaconazole, Ketoconazole		
IFI		Chemotherapy	voriconazole, amphotericin B, caspofungin, or posaconazole alone or in combination. The 2 patients with orbitocereb	All patients were given secondary prophylaxis with oral voriconazole, itraconazole, or posaconazole. The median time for secondary prophylaxis was 90 days (range: 39-429 days). Reactivation of IFI occurred in 4 patients as pulmonary IFI; all of them were cured completely after treatment	[28]
Mucormycosis		Chemotherapy	liposomal amphotericin B and posaconazole therapy in combination for a long duration.	Posaconazole has been reported to be effective in patients with mucormycosis who are refractory to or intolerant of liposomal amphotericin B or who need maintenance treatment	[29]
Candida		HSCT	Micafungin	Micafungin has shown to be effective in the treatment of IFI in patients with febrile neutropenia	[30]
Mold infections		HCST	Broad spectrum azoles	Genetically targeted antifungal prophylaxis would be particularly useful in patients undergoing intensive chemotherapy for acute leukemia, to whom universal prophylaxis is not always given, even though invasive aspergillosis remains more frequent in such patients than in solid organ transplant recipients.	[31]
IFI (Mucormycosis, candidiasis, cryptococcosis, and invasive aspergillosis)		Induction chemotherapy	L-AmB and	Patients with mucormycosis were successfully treated with a combination of surgery and prolonged antifungal treatment with L-AmB and posaconazole.	[32]
			Voriconazole,		
			Posaconazole		

In patients undergoing HSCT, the use of voriconazole has been observed to decrease IFIs, while fluconazole was responsible for breakthrough fungal infections. However, since azoles block cytochrome P-450 and thus slow the metabolism of vinca alkaloids, When ALL patients were treated with vincristine (vinca alkaloids), voriconazole was replaced by micafungin (echinocandins), which was also found to be effective in reducing IFI [20].

Invasive mold infections have significantly increased mortality in oncohematologic patients. Although prophylaxis is recommended, the associated problems of the considerable number needed to treat resistance and significant adverse effects prove to be concerning. Genetic testing for pentraxin-3 polymorphism has been associated with invasive mold infections among ALL patients undergoing chemotherapy [22]. Thus, genetic testing can be a possible way to curb invasive mold infections in ALL patients to whom universal prophylaxis isn’t always given.

Pneumocystis jirovecii is a ubiquitous fungus that usually affects immune-compromised individuals, such as the ones with HSCT, Chemotherapy, and malignancies, by usually causing Pneumocystis pneumonia (PCP). Guidelines suggest prophylaxis for six months post-transplant. It has been observed that the infection rate is 0.08% with trimethoprim/sulfamethoxazole and 0% to 1.3% with pentamidine as compared to 5% to 16% when no prophylaxis was given [25-27]. However, since PCP is relatively rare, and prophylactic drugs are associated with significant adverse effects; it should be used after carefully analyzing the risk-benefit. Rare cases of mucormycosis have been found in children undergoing the BFM-ALL protocol. The treatment is liposomal amphotericin B and posaconazole combined with surgical debridement [29].

Discussion

ALL affects the body's immune defense mechanism and weakens it by disallowing the differentiation of lymphocytes responsible for keeping microorganisms from causing infection. Furthermore, chemotherapy for ALL consists of high-dose systemic methotrexate, asparaginase, and dexamethasone, which significantly impacts the body's immune system primarily through myelosuppression. Infection is a significant cause of morbidity and mortality during treating children with ALL. Various bacterial, fungal, and viral pathogens can cause this.

The most common infections encountered in patients with ALL are viral infections, followed by bacterial infections and fungal infections. The most prevalent viral infections were found to be: CMV, AdV, and EBV. At the same time, coagulase-negative Staphylococcus species were found to be most common among bacteria. Currently, cefepime alone or Vancomycin-containing regimes control bacterial infections according to a study conducted in 2020 [33]. According to a study conducted in 2014, fluoroquinolones have been found to reduce bacterial infection in patients with ALL undergoing chemotherapy. However, the effect of fluoroquinolones on patients with ALL undergoing HSCT was negligible [25]. Another study conducted in 2015 concluded that levofloxacin can reduce the mortality and morbidity of pediatric patients with ALL [34].

The most common fungal infections were Aspergillus spp. (48%) and Candida spp. (38%). Invasive mold infections significantly cause mortality in oncohematological patients. Fungal infections are relatively lower [9], which may be attributed to primary prophylaxis with azoles and amphotericin B. Invasive mold infections seem to cause mortality in oncohematological patients significantly. Azoles and amphotericin B can be used for prevention; however, problems with high cost due to huge NNT, resistance, and significant adverse effects cause a hindrance. Genetic testing for pentraxin-3 polymorphism can detect patients likely to get invasive mold infections during the treatment for ALL [31].

Amongst the viral infections, the most commonly found organisms after HSCT are EBV, CMV, and AdV. Using serology to monitor CMV is necessary to start prophylaxis with antivirals. Ganciclovir is an effective drug for the prophylaxis and treatment of CMV infection. According to a study conducted in 2017, the HSCT recipient showed seropositivity, Ganciclovir was used for the prevention of CMV, rituximab for EBV, and cidofovir for AdV. CMV prophylaxis is especially required for transplant recipients with a seropositive CMV IgG [9].

Despite prophylactic treatment and inoculation of vaccines and immunoglobulins, infections are still common among ALL patients. Due to a limited number of studies conducted and published, the formulation of newly updated guidelines has not been successful. Specific tests like genetic testing are expensive to run. Poverty-related factors like low parental literacy, suboptimal living conditions, and lack of accessibility to healthcare in developing countries further hinder the care of infectious complications; thus, the aspect of increased financial burden and feasibility comes as a barrier [34]. Effective prevention and management of infections require timely access to diagnostics and antimicrobials by improving healthcare infrastructure.

## Conclusions

Due to the immunocompromised status of patients with ALL, they are more disposed to developing bacterial, viral, and fungal infections. Among all the infections these patients encounter, viral infections are most prevalent, followed by bacterial infections and fungal infections. Despite the inoculation of vaccines and the use of prophylactic treatments, the rate of development of infections in ALL patients is high. To decrease the risk of infections in ALL patients, it is required to reduce the duration of poor innate host defenses against infection, improve acquired immunity through vaccines or Igs, practice good hygiene, and use antibiotic prophylaxis for specific at-risk patients. Steps should be taken to overcome the challenges, including an insufficient number of healthcare personnel trained in infection care and prevention, inadequate microbiologic infrastructure, poor healthcare infrastructure, lack of basic medical supplies, equipment, and trained personnel, and insufficient institutional policies with poor organizational structure and forging public and private alliances to support cancer care. Furthermore, more research must be conducted to revise the guidelines frequently for effective treatment.
